# A 2020 synopsis of the cell-cultured animal industry

**DOI:** 10.1093/af/vfaa031

**Published:** 2020-10-30

**Authors:** Rhonda K Miller

**Affiliations:** Department of Animal Science, Texas A&M University, College Station, TX

**Keywords:** beef, cell-cultured protein, fish, pork, poultry

**Implications**

- The cell-cultured animal industry is marketing consumer available products in 1 to 5 yr, and time to market is dependent on development of technologies, regulatory approval, and product structure (intact vs. non-intact).
- Initial products will be higher priced than traditional products and most likely sold in upscale markets.
- By 2050, cell-cultured companies anticipate localized production facilities throughout the world producing cell-based meat, poultry, and seafood products to assist in supplying the demand for protein.

## Introduction

Commercialization of cell-cultured meat products, is it fact or fiction? Will the cell-culture industry provide sufficient product to help feed the projected population growth by 2050? While there is not a crystal ball to answer these questions, the cell-cultured animal industry is evolving and making strides toward commercialization of protein-based meat, fish, and poultry products. Cell tissue cultures have been used in research to understand basic muscle tissue growth, impact of disease states on tissue growth and development, and development of medical treatments. This research is conducted in sterile laboratory conditions and has brought advancements that have benefited animals, plants, and humans. This research technology is now being scaled up to develop protein products for human consumption (Post, 2012; Stephens et al., 2019). While extensive media coverage has helped to inform the animal and meat industry of this trend, multiple questions on the state of development and products that may be produced are not known. The objective of this paper is to provide an overview of the proposed commercial cell-cultured meat production process, and to provide insight from the companies and industry that are developing cell tissue proteins for commercial production.

## An Overview of Commercial Production of Cell Tissue Culture

**Figure 1.**
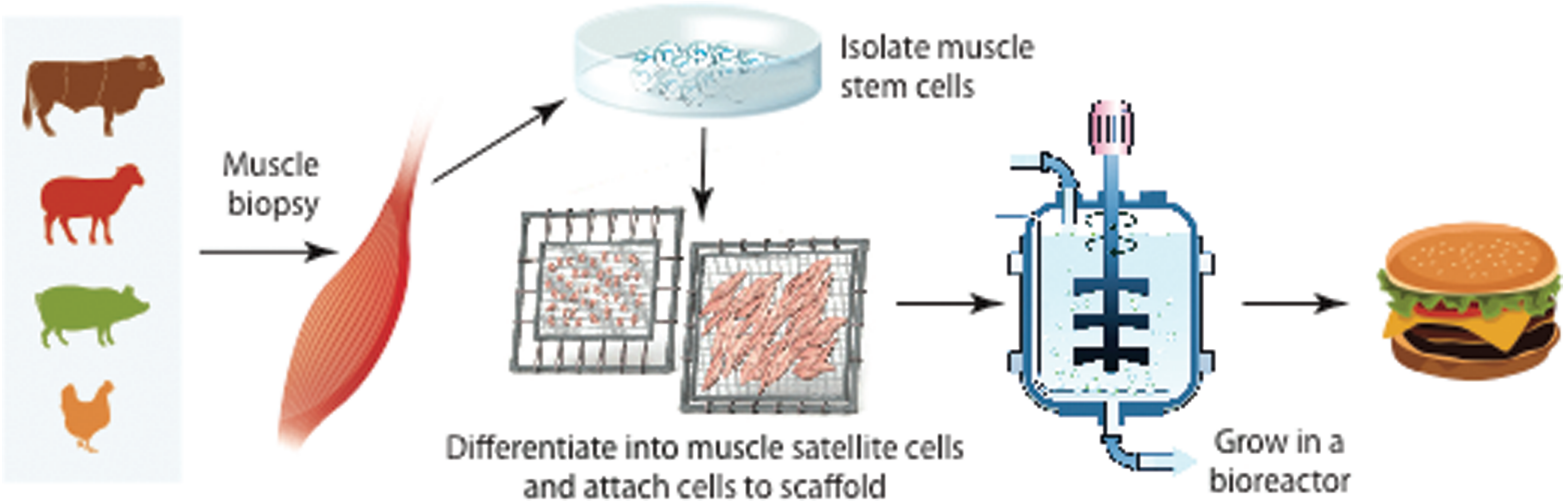
A simplified schematic of production of cell-cultured protein. Potential photos to be used in the article from companies websites.

The production of cell-cultured protein products is the scaling up of the cell culture meat production process used in biological research since the 1970s (Ben-Arye and Levenberg, 2019). A simplified schematic of cell-cultured protein production is provided in Figure 1. The basic process uses starter cells that are obtained from animals harvested with biopsy techniques or are from immortalized cell lines, among other sources. Satellite cells (specialized cells within muscles that can replicate) that are harvested from biopsies or are satellite cells from immortalized cell lines are used. Satellite cells are then placed in small cell cultures to grow and multiply. The cells are grown on material called scaffolding. Currently, edible forms of scaffolding are being developed. The scaffolds with cells attached are placed in sterile tanks called bioreactors in which nutrients for growth are provided. The conditions or environment required for cell growth and proliferation are provided. Additionally, growth medium, either fetal bovine serum or formulated serum that is species specific, other nutrients, water, and physical stimulus at temperatures conducive for cell growth are also included. Cells grow, multiply, and develop into mature muscle cells. Depending on the initial fate of cells, connective tissue and adipose tissues either develop with the muscle cells or are added. When harvested, the cells are very thin pieces of tissue. The thin layers of cells can be processed by layering the thin pieces into an intact meat product or ground to produce comminuted meat products.

The key in this production scheme is that the species of the original biopsy or cell line determines the species of origin of the final product. Additionally, a high level of sanitation and the limiting of cross-contamination of materials during the production phase are critical to obtaining cells that proliferate and grow to their potential. The industry, and particularly private companies, has made great strides in understanding the production conditions, inputs and processing needs to produce cell-cultured protein, yet full commercialization has not been obtained as of May 2020. Although some products have been highlighted in the media, products have not been commercialized to date. The industry is in the development stage and is examining scale-up challenges. Issues include, but are not limited to, the development of scaffold materials that are edible or re-usable, decreased cost for growth medium as fetal bovine growth serum is expensive and limited in supply, and environmental and exercise conditions needed for maximum muscle growth. While this industry is developing, questions are being asked about the meat science challenges and when the industry will be ready to market products.

## Meat Science Challenges

While the production of cell-cultured protein may appear well defined, there are several challenges facing this industry from a scale-up and meat science standpoint. These challenges were presented at the meetings discussed below in 2018 by the American Meat Science Association. While progress has been made to answer these questions, there has been limited to no product available for evaluation. Biotechnology companies continue to work toward development of their process and to answer product quality, functionality, and microbiological questions. Some of the meat science issues and questions about the classification of cell-cultured products as meat that have been discussed are how the conversion of cells into meat occurs. Meat contractile state, pH, and color are affected by the conversion process and the subsequent shelf-life, consumer acceptance, and meat palatability are influenced by these conversion factors. How muscle cells go through conversion of muscle to meat when removed from the life supporting environment is important for consistent meat appearance, functionality, eating quality, and shelf-life. Meat pH and consistency of meat pH has been extensively studied and it has been well established that meat pH is related to meat color and microbial growth. Lower than normal final pH, generally less than 5.4, results in light colored meat with low water holding capacity and high final pH, generally greater than 6.0, results in darker meat with high water holding capacity and limited shelf-life due to increased conditions for microbial growth. Additionally, final meat pH influences meat color, consistency in meat color and consumer acceptability. Consumers purchase meat first based on visual appearance. Consumers have indicated that meat color is their indication of freshness or shelf-life. Meat that is either two light or too dark within species has reduced consumer acceptance. Additionally, meat that is two toned or has variation in color along the lean surfaces is unacceptable in visual appearance. In meat with variations in pH, color will vary consistent with differences in pH. pH also affects microbiological shelf-life. Maintenance of consistent pH throughout a meat product affects microbiological growth. Another major factor influencing meat shelf-life is cross-contamination during slaughter, fabrication, and processing. In conventional animal production at harvest, muscle from healthy animals is free of bacteria. In conventional animal production, cross contamination of microbial hazards may occur on the outside of the meat during harvesting and processing, with the interior being essentially sterile. Initial microbiological levels, meat pathogen levels, temperature, environmental conditions, and pH greatly influence length of storage prior to spoilage and meat microbial pathogen growth. Questions concerning if meat spoilage and pathogenic microorganisms growth and proliferate similarly in cell cultured and conventionally produced meat will need to be addressed. The meat industry uses intervention strategies during slaughter, fabrication, and processing. Interventions are used to reduce microbial levels and meat pathogens on the surface of whole muscle meat and/or within the product for non-intact or processed meats. Current interventions used in conventionally produced meat may not be the same as for cultured animal tissue. The challenges in elimination of microbial hazards in cultured animal tissue may be different than in animal production systems. As cell-cultured tissue is produced as thin layers of tissue and then combined, potential for cross-contamination is different than in animal systems. It is logical to hypothesize that the potential for introduction of hazards may occur in both systems, but different interventions may be needed and validated. While microbial growth of spoilage organisms are a major factor in product shelf-life, color stability, myoglobin content, and other conditions may affect shelf-life or sell-by dates of these products at retail. Additionally, as cooked internal temperature guidelines differ for non-intact vs. whole muscle meat and for meat from different species, issues influencing cook guidelines will need to be vetted. As product is not available for testing, when product production systems are intact, these questions can be examined.

There are other questions for cultured protein tissue across species concerning nutrient content. Muscle from conventionally raised farm animals, poultry, and fish provide energy intake and essential nutrients such as protein, lipid and trace mineral, and vitamins to the human diet (De Smet, 2012). In the live animal, nutrient content of meat, poultry, and fish is regulated by nutrient intake, digestive mechanisms, and the biochemistry of muscle. Nutrient content in muscle is highly regulated through homeostatic mechanisms throughout the animal system. Van Paemel et al. (2010) reviewed 27 micronutrients in meat and linked how concentrations in the meat were affected by animal dietary intake. However, while the nutrient and micronutrient content in the growth medium for cell protein tissue growth will be highly regulated to maximize cell growth, nutrient, and micronutrient content will need to be validated. It is reasonable to project that without the homeostatic mechanisms of the whole animal, content of some nutrients and micronutrients may differ. When cell-cultured tissue is available, an understanding of the nutrient, micronutrient, and amino acid content, nutritional bioavailability, and protein functionality when compared with traditionally raised meat will need to be compared. Additionally, research on how cell-cultured tissue proteins function during processing, cooking, curing, smoking, drying, and/or fermenting and their subsequent variability in these attributes also will be needed.

Meat eating quality and consistency in eating quality are critical issues for the cell-cultured protein industry. Consumer acceptance, while initially based on meat color and labeling, has been shown to be affected by sensory characteristics and consistency in sensory characteristics for repeat purchases. The flavor and texture attributes of cell-cultured tissue and how it compares to conventionally produced meat needs to be addressed when cell-based meat is available for evaluation. Consumer acceptance for the technology is another question. An understanding of consumer segments and the drivers of acceptance for cell-based cultured protein products will be needed. Companies in the cell-cultured protein industry have stated that this production system is more sustainable and has lower water and antibiotic usage compared with traditional production (Lynch and Pierrehumbert, 2019). As the technology develops, an understanding of sustainability, water usage, and antibiotic use will need to be evaluated.

While this in an overview of the meat science questions in the commercialization of cell-cultured protein production, other questions may arise. The commercialization of the industry is in the development stage. Multiple companies are developing production systems and some of the first questions were which government agencies would have responsibility for regulation and oversight.

## Oversight and Regulation

The US Department of Agriculture, Food Safety and Inspection Service (**FSIS**) and Department of Health and Human Services Food and Drug Administration (**HHS-FDA**) held two meeting in 2018 to discuss the current state of the cultured meat industry and listened to industry feedback. On March 7, 2019, HHS-FDA and USDA-FSIS announced a formal agreement to jointly oversee the production of human food products derived from the cells of livestock and poultry (USDA, 2019). In this agreement, HHS-FDA would have oversight of tissue collection, cell lines and banks, and all components and inputs. They would be involved in premarket consultation processes to evaluate production materials/processes and manufacturing controls, and they would share these results with USDA-FSIS. As a component of this responsibility, HHS-FDA would oversee the proliferation and differentiation and maintenance of cells and cell banks, and help to transfer the regulatory oversight to USDA-FSIS. In conducting these responsibilities, HHS-FDA would be responsible for appropriate inspections. The USDA-FSIS would take over responsibility for the assurance of safety for cultured cell tissue production at harvest for foods that will have the USDA mark of inspection. These food items will be subject to inspection where these products are harvested, processed, packaged and labeled and will be subjected to the applicable FSIS regulations. These regulations include sanitation and physical product inspection, Hazard Analysis and Critical Control Point (**HACCP**) verification, product testing and records review. The USDA-FSIS will conduct oversight to assure that the resultant products are safe, unadulterated, wholesome, and properly labeled as is their responsibilities for meat and poultry production. The two agencies will work together so that HHS-FDA and USDA-FSIS do not overlap responsibilities but communicate information from one segment of production to the other.

This agreement provided a roadmap for moving this industry forward and utilizing the expertise and strengths of each agency. With the initial issues of regulation and oversight, the path was opened up for the cell culture industry to produce product. BlueNalu, Finless Foods, and Memphis Meats have started the regulatory process. As both companies are in the fish industry, regulation and label approval will be with FDA. Companies in the meat and poultry area have not, to date, obtained label approval where the combination of FDA and USDA-FSIS are used. While both regulatory agencies have been proactive in addressing how oversight of the cell-cultured industry will occur, the process has not been completed through the steps of HACCP approval and a label has not been awarded.

## Where Is the Industry?

**Figure 2.**
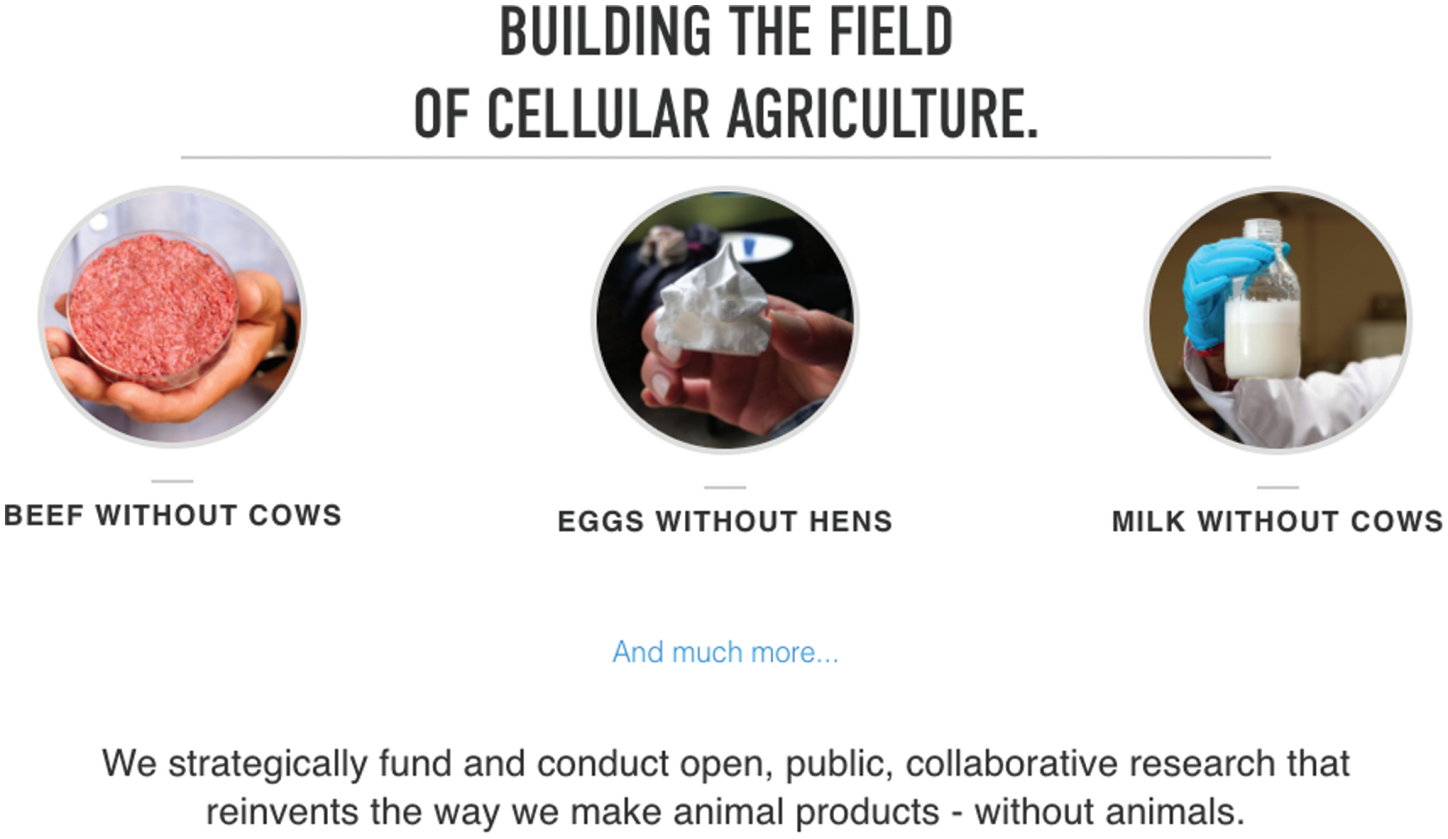
New Harvest is building the field of cellular agriculture (*Source*: new-harvest.org; accessed June 29, 2020).

To understand the state of the industry, companies that are actively developing cell-cultured protein products were contacted (Table 1). While not every company that is developing cell-cultured meat for protein consumption was contacted, major companies were included to obtain a general industry understanding and projection for the future. Most of these companies attended the 2018 meetings of HHS-FDA and USDA-FSIS. Additionally, industry expert and consultant Dr. Paul Mozdziak, Professor with the Prestage Department of Poultry Science at North Carolina State University provided his insight into the developing cell-culture industry. Individuals at two major industry organizations, Dr. Kate Krueger at New Harvest (https://www.new-harvest.org/) a 501(c)(3) research institution assisting in development of cellular agriculture and Cathy Cochran, Vice President at GPG talked about AMPS Innovation (AMPS Innovation; https://ampsinnovation.org/), a coalition of food companies dedicated to development of protein-based foods from animal cells (Figure 2). Insight into the current state of industry development, into potential products and species-specific products being developed, on consumer segments targeted, vision on potential impact of this technology in the next 50 yr, and any additional information on the scope of the industry was discussed.

**Table 1.** Summary of cell cultured protein companies that contributed

Company	Country/industry role and/or targeted species	Product
BlueNalu	US/fish	Mahi–mahi fillets
Finless Foods	US/fish	Blue fin tuna fillets
Memphis Meats	US/beef, poultry, pork, and other species	Meat, poultry, and fish whole muscle and restructured products
New Harvest	US/nonprofit research institution dedicated to accelerating the pace of innovation in cellular agriculture	
Alliance for Meat, Poultry and Seafood Innovation	US/alliance of cell-cultured-based companies; role is education, advocacy for policies, and programs for pathway to market cell-based/cultured meat products	
Aleph Farms	Israel/beef whole muscle product where muscle and adipose cells grow simultaneous with connective tissue structure and growth	Beef whole muscle products; future will examine lamb and pork

The cell-cultured protein industry is a global industry. Globally, companies are actively developing systems and products (Table 2). While the popular press has indicated that product will be commercially available soon, the general theme by companies in this space was that products are at least 1 to 3 yr from active commercialization. Some individuals indicated that they projected 2 to 3 yr and maybe up to 5 yr before products are readily available depending on whether they were directing production to structured, or intact muscle protein products, or unstructured defined as ground/comminuted protein products. Dr. Paul Mozdziak indicated that cell-cultured protein companies are in the stage of innovation and development of these companies are occurring in the biotechnology arena. The biggest challenges that companies face are to develop the cell culture process to scale and deliver a product at an acceptable price point. Dr. Kate Krueger, Research Director at New Harvest indicated that, while it is hard to know when products will be available, it is expected that it will take 10 to 15 yr for intact muscle products to be commercialized in volume for consumers in the retail food space at competitive prices (personal communication, Dr. Kate Krueger, New Harvest). Krueger also indicated that the industry expects unstructured meat products to be commercialized first. Products mimicking chicken nuggets, ground beef, sausages, and frankfurters will most likely be on the market prior to whole muscle poultry, beef, and pork products.

**Table 2.** Summary of additional cell-cultured protein companies and organizations as of June 2020

Company	Country/industry role and/or targeted species	Product
Higher Steaks	United Kingdom/pork	Sausage and bacon
Shiok Meats	Singapore/seafood and meats	Shrimp, crab, and lobster
Future Meat Technologies	Israel/proprietary technology for growing fibroblast cells	
Wild Type	United States/fish	Salmon, minced, and filets
Just	United States/chicken and beef	Chicken nuggets, and Wagyu beef
Mission Barns	United States/fat cells	Pork/bacon fat and duck fat
New Age Meats	United States/pork	Pork sausage
Wild Earth	United States/high protein fungus	Pet food
BioFood Systems	Israel/beef	Beef
MeaTech	Israel/3D stem cell printing technology	Chicken
Super Meat	Israel	Chicken
Balletic Foods	US	
Bond Pet Food	US/DNA from a heritage hen	Pet food
Meatable	The Netherlands/using OPTi-OX	
Mosa Meats	The Netherlands/first hamburger patty in 2013	Beef
Appleton Farms	Canada	Beef
Future Fields	Canada	Chicken
SeaFuture	Canada	Fish
IntegriCulture	Japan/duck/	Foie gras
Nissin Foods Group	Japan/beef/	Diced steak
BioTech Foods	Spain/pork/	Minced meat for sausages and meatballs
Cubiq Foods	Spain/animal fat	Fat enhanced with omega-3 fatty acids
Gourmey	France/duck	Foie gras
Heuros	Australia	Developing cell culture medium for other companies
VOW	Australia	Kangaroo
Avant Meats	China/fish	Fish maw
Peace of Meat	Belgium/multiple species	Foie gras and chicken
Ochakov Food Ingredients	Russia	Beef; meatloaf
Craveri Laboratories	Argentina	Unknown but imply beef
Biftek	Turkey	Beef
Clear Meat	India/chicken kheema biryani	Minced chicken

**Figure 3.**
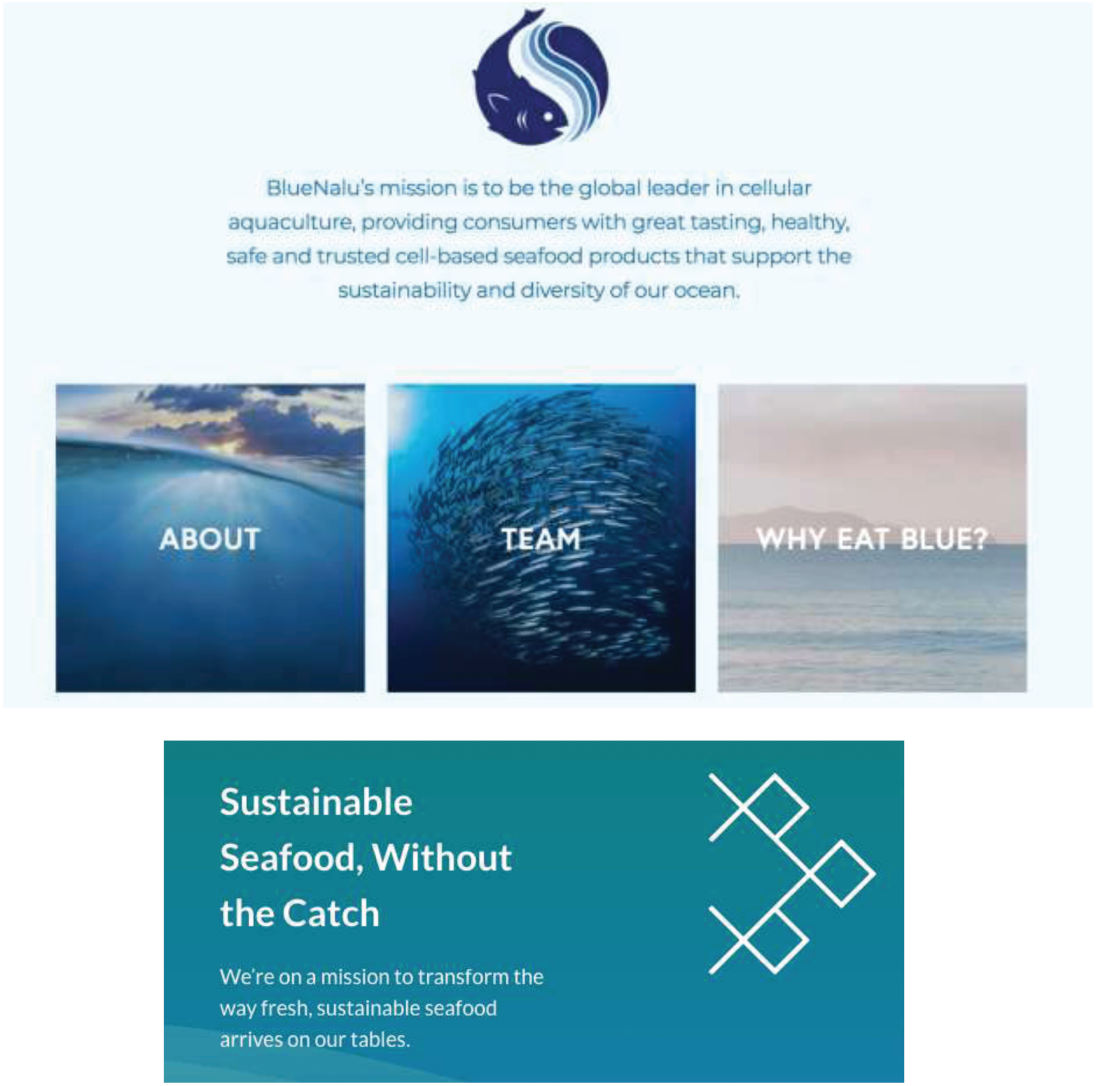
Several companies have targeted fish or seafood products including BlueNalu (top, *Source*: bluenalu.com; accessed June 29, 2020) and Finless Foods (bottom, *Source*: finlessfoods.com; accessed June 29, 2020).

**Figure 4.**
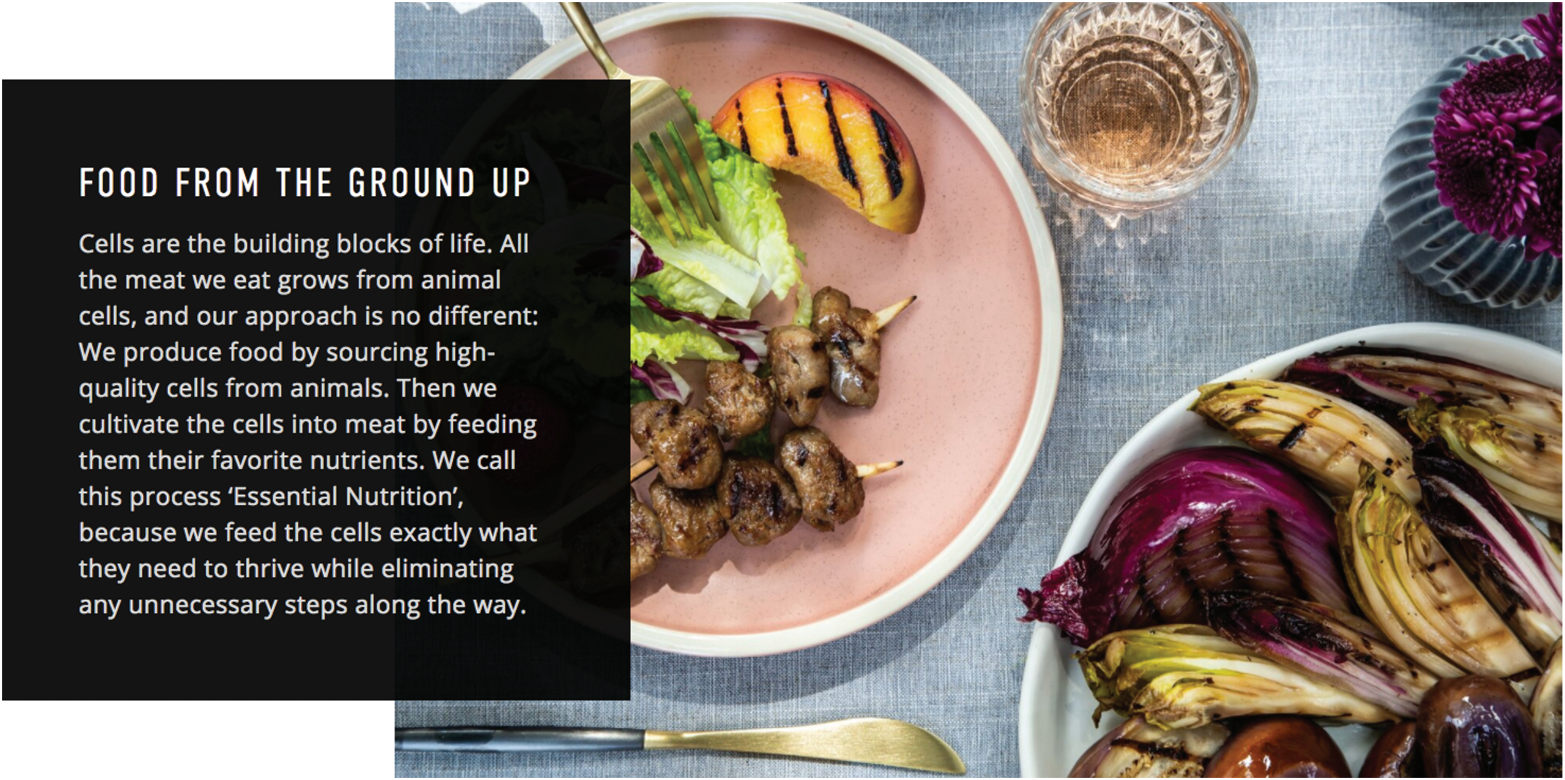
Memphis Meats is building a production platform for many types of meat (*Source*: memphismeats.com; accessed June 29, 2020).

Companies developing fish products, BlueNalu and Finless Foods, appear to be closer to commercialization than companies developing beef, pork, and poultry products (Figure 3). Dr. Krueger stated that development of protein using insect and crustacean cells may be closer to marketing than protein products from other species (personal communication, Dr. Kate Krueger, New Harvest). BlueNalu (www.bluenalu.com/) is targeting production of mahi–mahi fillets and Finless Foods (www.finlessfoods.com) is directing their effort toward production of Blue Fin tuna fillets. They have targeted this species as mahi–mahi cannot be produced using aquaculture technology or as classified as farm-raised seafood. Mahi–mahi fillets merchandized from the live catch industry. Mahi–mahi are aggressive fish and will kill each other if placed in close proximity as in farm-raised production systems. This provides BlueNalu the ability to provide mahi–mahi fillets to the consumer where there is currently a limited supply or a supply gap. BlueNalu is working with FDA on plant plans, HACCP plans, and other regulatory issues (personal communication, Lou Cooperhouse, BlueNalu). BlueNalu has identified ocean health, ecosystem health, community health, animal welfare, and personal welfare as reasons for developing the industry. Memphis Meats (https://www.memphismeats.com/) who showcased the first cell-based beef meat ball in 2016 and the first cell-based chicken and duck in 2017 is building a production platform that can produce any commonly eaten type of meat (personal communication, David Kay, Memphis Meats). Memphis Meats is targeting the production of both structured and unstructured meat products (Figure 4).

**Figure 5.**
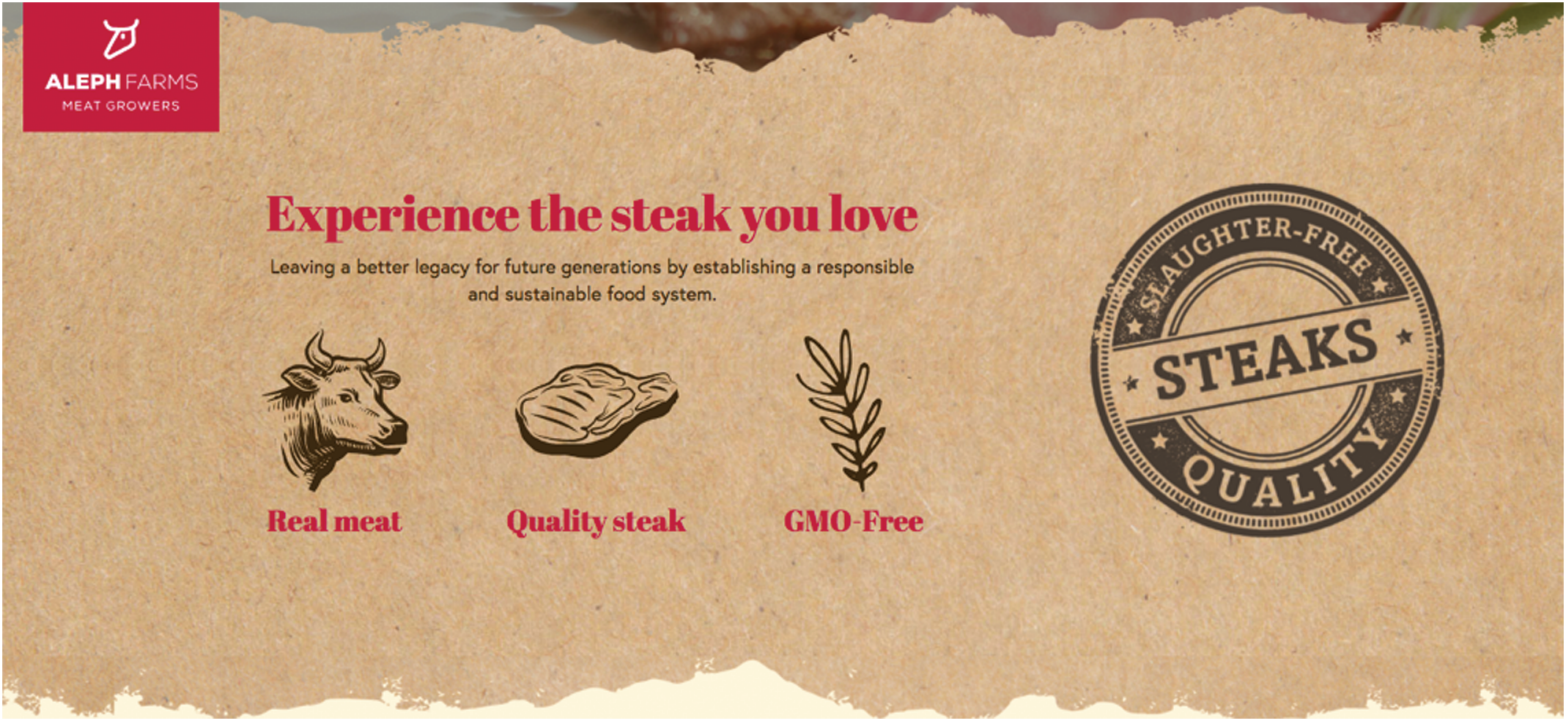
Alpeph Farms plans on co-culturing many cell types in concert to more closely mimic complex tissues (*Source*: alpeph-farms.com; accessed June 29, 2020).

Aleph Farms was established 2.5 yr ago in Israel, and they are taking a slightly different approach to production of cell-cultured products (personal communications, Neta Lavon, Aleph Farms). Aleph Farms are using technology developed at the Israel Institute of Technology to scale up the production of whole muscle beef where stroma cells, adipocytes, and muscle fibers grown in concert similar to living tissue. Ben-Arye and Levenberg (2019) discuss how multiple cell types can be co-cultured on a 3D scaffold that will generate muscle fibers, blood vessels, and a dense extracelluar matrix or connective tissue. Aleph Farms defined this uniques approach to cell-cultured product as alternative meats (Figure 5). The company does not see their technology replacing livestock production, but they perceive working hand in hand with the livestock, grain, and cell-cultured industries. Neta Lavon (www.aleph-farms.com) defined their company and technology as additive to current grain and livestock production. They are working with the grain industry for the development of 3D scaffolding for their production. In the future, they see bioreactors on farms that are producing the grains or plant materials so that production would take place on-farm. They called these farms Biofarms or Technological Farms.

Companies (Table 1) had general consensus that development of the industry will be a stepwise approach. The first products produced will be higher priced and targeted to specialty markets in affluent urban areas. As the industry develops and has time to examine cost reduction strategies, the cost of the product will decrease. Companies are currently addressing production scale-up methods and cost reduction for growth media strategies. As these companies are currently in the development stages, it is anticipated, as with any industry, that cost reduction and system efficiency technologies will continue to be addressed by research and development personnel especially after initial production of product. As the companies develop and place initial products into production, decreased cost reduction strategies will eventually assist them in expanding their market from specialty, premium products to pricing that is more similar to commodity-based products. How long will this take? Companies indicated from 3 to 7 yr depending on market success.

The big issue companies and professionals discussed were consumer acceptance. Consumer acceptance has not been sufficiently vetted; however, consumer surveys indicate that the more familiar consumers are with the concept, the more enthusiastic they about potential purchases. Siegreist (2008) talked about how important consumer acceptance was for success of new technologies. While products will initially be marketed in urban hubs, it is anticipated that access to these products would grow consumer awareness and move the product to have higher demand with continued production and introduction into the market (personal communication, Shannon Cosentino-Roush, Finless Foods). Dr. Bill Hallman, Professor and Chair of the Department of Human Ecology at Rutgers University and School of Environmental and Biological Sciences conducts research on public perceptions of controversial issues concerning food, health, and the environment. He is working with BlueNalu in understanding consumer perception for cell-based protein products. While these research results are not public to date, results will be forthcoming. Research to examine consumer attitudes for cell-cultured meat has been conducted in multiple populations (Vanhonacker et al., 2013; Verbeke et al., 2015, 2015; Bekker et al., 2017). Bryant and Barnett (2018) reviewed research that has been conducted on consumer acceptance of cultured meat mainly in Europe and the United States. They found that there were multiple factors influencing acceptance as well of objection to cultured meat. It is apparent that there are consumer segments toward attitudes on cell-cultured meat. While cell-cultured meat may not be universally accepted, there are consumer segments that have indicated a willingness to purchase these products.

For long-term goals beyond the 2 to 5 yr goals discussed above, companies indicated that in 30 to 50 yr they would expect cell-cultured protein products to be readily available to consumers in the foodservice and retail markets. Ideally, they would like to have localized production. Facilities would be located in local markets that would produce protein products for that area. It is anticipated that locations would range from urban hubs in affluent and emerging economies and/or in areas where protein production is limited based on environment, soil, or economic factors. The facilities would also contribute to the local economy by providing additional employment opportunities even though the requirement of labor would not be as great as current meat processing facilities. Additionally, companies indicated that smaller plants placed locally would have increased food security, a current food industry concern and issue while providing improved nutrition through lower priced, available protein products.

While not an aspect for cell-cultured proteins that will be initially marketed, individuals indicated that cell-based products may be a basis for carrying important nutritional components for humans. For example, increased levels of omega fatty acids or nutrients that are naturally low in the human diet could be engineered into these products in the future. While consumer acceptance of the technology is the first step for success of this industry, the addition of nutrients for health was indicated as a step for the future as it is easier to increase nutrients in a cell-cultured system than in the live animal. Danielle Beck at the National Cattlemens’ Beef Association indicated that consumer acceptance will be key to this industry’s success (personal communication, Danielle Beck, National Cattlemens’ Beef Association). They have seen consumers accept and reject new technologies, so positioning the initial products in the marketplace will be critical to the industries success and sustainability.

In anticipation of greatly increased demand for quality protein by 2050 as the global population is projected to reach 10 billion, the cell-cultured protein companies see that they have a role to helping to feed the world. David Kay from Memphis Meats said that given the amount of resources required to produce meat from animals, there are not enough resources on the planet to meet that growing demand (personal communication, David Kay, Memphis Meats). Cell-based meat can, alongside conventionally produced meat, enable food systems to meet that demand for meat, while also preserving the environment and society’s most cherished culinary traditions (personal communication, David Kay, Memphis Meats). Danielle Beck, at the National Cattlemens’ Beef Association indicated that as global food demand increases, the beef industry sees the need to work together with the cell-cultured protein industry as part of the solution to meet consumer protein needs (personal communication, Danielle Beck, National Cattlemens’ Beef Association).

## Conclusions

This is a dynamic industry that is rapidly developing. While exact time to commercialization is unknown, most likely products initially will be available for consumer purchase in the next 1 to 3 yr with increased product availability through the next 10 to 30 yr. The aquatic protein-based companies are expected to have the first product on the market followed by other protein sourced products. The regulatory framework is being put into place and the industry will have to adhere to food safety, labeling, and production oversight as their counterpart commodity-based industries. First on the market products will most likely be higher priced than their commodity-based counter parts and with time and cost reduction strategies, prices will decrease. While cell-cultured protein products will initially be targeted toward higher end markets and restaurants, as price is reduced more wide scale distribution is expected. For some companies, placing production plants in geographical locations where protein production is limited is an ultimate long-term goal. The most important question that cannot be answered is how consumers will accept these products. While exposure to the product and education will help in the acceptance of the product, long-term acceptance is not known and will be driven my multiple factors. While consumers accept new technologies, they also reject some technologies. Work on consumer attitudes is being conducted and as products become available on the market, understanding consumer purchasing behaviors will become paramount and will either drive or limit the success of this industry segment.

Initially, it was perceived that this industry would reduce consumption of conventionally produced meat and compete head on head. After discussion with individuals working in this segment of the industry, it is apparent that, while eventually there may be some competition, the cell-cultured protein industry will be providing a product for specialty markets and upscale or sustainability-minded consumers in the short term. As the world population grows, the cell-cultured protein industry may provide avenues to locally produce protein especially in areas where livestock production is limited due to environment and underlying economy. Development of this industry in ultimately in the consumer’s hands. Consumer acceptance will drive the success of these products.

*Conflict of interest statement.* None declared.

## About the Author

Rhonda Miller earned a BS (1978), MS (1982), and Ph.D. (1983) in Animal Science from Colorado State University. In 1983, she became the Director of Research and Development for Monfort, Inc., and in 1988, she joined the Animal Science faculty at Texas A&M University. Currently, she is a Professor, AgriLife Faculty, and Meat Science Fellow. She has taught meat science courses and developed a research program examining the palatability, composition, and shelf-life of meat products. She is a recognized meat sensory scientist, has won extensive awards for teaching and research, served on key industry committees, and was the Director and President of AMSA.
